# Combinatory effect of BRCA1 and HERC2 expression on outcome in advanced non-small-cell lung cancer

**DOI:** 10.1186/s12885-016-2339-5

**Published:** 2016-05-14

**Authors:** Laura Bonanno, Carlota Costa, Margarita Majem, Jose-Javier Sanchez, Ignacio Rodriguez, Ana Gimenez-Capitan, Miquel Angel Molina-Vila, Alain Vergnenegre, Bartomeu Massuti, Adolfo Favaretto, Massimo Rugge, Cinta Pallares, Miquel Taron, Rafael Rosell

**Affiliations:** Medical Oncology 2 Unit, Istituto Oncologico Veneto I.R.C.C.S, Via Gattamelata 64, 35128 Padova, Italy; Laboratory of translational Oncology, Pangaea Biotech, Sabino de Arana, 5-9, Barcelona, Spain; Medical Oncology Service, Hospital de Sant Pau, Sant Antoni Maria Claret, 167, Barcelona, Spain; Autonomous University of Madrid, Ciudad Universitaria de Cantoblanco, 28049 Madrid, Spain; Department Obstetrics, Gynecology and Reproduction, Dexeus Universisty Hospital, av Sabino de Arana 5-9, Barcelona, Spain; Hospital du Cluzeau, 23, rue Larey, Limoges, France; Medical Oncology, General Hospital of Alicante, 11, Baeza, 03010 Alicante, Spain; Cytology and Pathology, Università degli Studi di Padova, Via Gabelli 61, Padova, Italy; Catalan Institute of Oncology, Barcelona, Spain

**Keywords:** BRCA1, HERC2, Non-small-cell lung cancer, Platinum, Predictive markers, DNA repair

## Abstract

**Background:**

BRCA1 is a main component of homologous recombination and induces resistance to platinum in preclinical models. It has been studied as a potential predictive marker in lung cancer. Several proteins modulate the function of BRCA1. The E3 ubiquitin ligase HERC2 facilitates the assembly of the RNF8-UBC13 complex to recruit BRCA1 to DNA damage sites. The combined analysis of multiple components of the pathway leading to the recruitment of BRCA1 at DNA damage sites has the potentiality to improve the BRCA1 predictive model.

**Methods:**

We retrospectively analyzed 71 paraffin-embedded tumor samples from advanced non-small-cell lung cancer patients treated with first-line platinum based chemotherapy and measured the mRNA expression levels of *BRCA1*, *RNF8*, *UBC13* and *HERC2* using real-time PCR. The mRNA expression was categorized using median value as cut-off point.

**Results:**

The median progression-free survival of all 71 patients was 7.2 months whereas the median overall survival of the study population was 10.7 months. Among patients with low *BRCA1* expression, the median PFS was 7.4 months in the presence of low *HERC2* levels and 5.9 months for patients expressing high *HERC2* levels (*p* = 0.01). The median OS was 15.3 months for patients expressing low levels of both genes and 7.4 months for those with low BRCA1 but high HERC2 (*p* = 0.008). The multivariate analysis showed that among patients with Eastern Cooperative Oncology Group performance status 0–1, the combined low expression of both *BRCA1* and *HERC2* clearly reduced the risk of progression (*p* = 0.03) and of death (*p* = 0.004).

**Conclusions:**

These findings confirm the potentiality of integrated DNA repair components analysis in predicting the sensitivity to platinum in lung cancer. The study indicates a predictive role for HERC2 mRNA expression and paves the way for further refinement of the BRCA1 predictive model.

**Electronic supplementary material:**

The online version of this article (doi:10.1186/s12885-016-2339-5) contains supplementary material, which is available to authorized users.

## Background

Platinum-based chemotherapy is currently the first-line treatment of choice for patients with advanced non-small-cell lung cancer (NSCLC) in the presence of wild-type epidermal growth factor receptor and non-rearranged ALK. However, no reliable predictive biomarkers of platinum resistance are currently available for routine clinical use. Breast cancer susceptibility gene 1 (BRCA1) plays a pivotal role in the repair of platinum-induced DNA damage and has been associated with cell resistance to platinum in preclinical models [1–3]. From the biological point of view, BRCA1 is involved in two main mechanisms of repair of platinum-induced DNA damage. The first one is nucleotide excision repair (NER), being the main pathway for the repair of helix-distorting DNA lesions [4, 5]. The second one is homologous recombination, an error-free mechanism for the repair of DNA double-strand breaks [6].

In clinical retrospective series, low mRNA expression of *BRCA1* was associated with longer survival in NSCLC patients treated with cisplatin-based neoadjuvant chemotherapy [7], while the clinical feasibility of prospectively assessing *BRCA1* mRNA expression was later demonstrated in a prospective phase II trial in advanced NSCLC patients [8]. Despite these encouraging preliminary results, a phase III randomized trial (NCT00617656/GECP-BREC) comparing non-biomarker-directed therapy with treatment based on the mRNA expression levels of *BRCA1* and *receptor-associated protein 80 (RAP80)* was recently closed prematurely, since the interim analysis showed a detrimental effect in terms of progression free survival (PFS) in patients allocated to the experimental arm (hazard ratio [HR], 1.35; *p* = 0.03) [9, 10]. The protein RAP80 is a component of one of the BRCA1 complexes at DNA damage sites and may be essential in the assembly of the BRCA1-A complex at DNA damage sites [11]. Retrospective analyses had demonstrated that *RAP80* mRNA expression could affect the predictive capacity of *BRCA1* [8, 12].

**Fig. 1:**
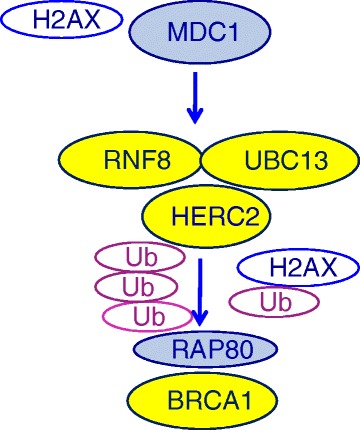
The protein BRCA1 is recruited at DNA damage through a recognition mechanism implying the phosphorylation of histone H2AX and leading to the assembly of a protein complex that induces histones post-translational modification called ubiquitination. This protein complex includes the RING finger ubiquitin ligase 8 (RNF8), the conjugating enzyme 13 (UBC13) and the E3 ubiquiting ligase HECT domain-containing protein 2 (HERC2). The biological model of BRCA1 recruitment at DNA damage sites was used to select the genes to evaluate as potential predictive markers of platinum sensitivity in lung cancer

However, several other DNA repair components are required for the recruitment and the function of BRCA1 in DNA repair [13, 14]. In particular, post-translational protein modification called ubiquitination is fundamental for the assembly of effector proteins complexes. After the phosphorylation of mediator of DNA damage checkpoint 1 (MDC1), RING finger ubiquitin ligase 8 (RNF8) is recruited at DNA damage sites and creates a complex with ubiquitin conjugating enzyme 13 (UBC13). This complex induces the formation of Lys 63-linked ubiquitin chains, which are essential for the assembly of BRCA1 complexes at double-strand breaks [15–17]. Recently, HECT domain and RCC1-like domain-containing protein 2 (HERC2), which functions as an E3-ubiquitin ligase, was shown to facilitate the formation of the RNF8-UBC13 complex [18] (Fig. 1). The protein HERC2 is also involved in regulating the stability of BRCA1 at DNA damage sites, since HERC2 ubiquitinates BRCA1 and targets it for degradation when BRCA1 is not in a complex with BRCA1-associated RING domain protein 1 (BARD1). The BRCA1-BARD1 heterodimer is required for BRCA1 stability, nuclear localization and E3 ligase function [19, 20]. This role of HERC2 is enhanced in the S-phase of the cell cycle, thus contributing to the modulation of BRCA1 function throughout the cell cycle and to the role of BRCA1 at the G2-M checkpoint [21].

On the basis of this biological model, we hypothesized that RNF8, UBC13 and HERC2, being the main protagonists of ubiquitination process leading to BRCA1 recruitment at DNA damage sites, could modulate the predictive model based on BRCA1 expression.

## Methods

We retrospectively analyzed a series of 71 patients diagnosed with advanced NSCLC and treated with cisplatin or carboplatin plus gemcitabine or pemetrexed in the first-line setting (Table 1). Patients were selected according to first-line treatment, not including taxanes or vinca alkaloids, and availability of adequate tumor samples. No further clinical selection was performed. Radiological response was assessed using the Response Evaluation Criteria for Solid Tumor (RECIST) v 1.0 [22]. Progression free survival (PFS) was calculated from the beginning of first-line treatment until demonstrated radiological progression or death from any cause. The overall survival (OS) was calculated from the start of platinum-based chemotherapy to death from any cause.

**Table 1:** Characteristics of 71 patients with advanced non-small-cell lung cancer

	N (%)
Age median (range)	63 (40–82)
Gender	
Male	54 (76)
Female	17 (24)
ECOG PS	
0	9 (13)
1	44 (62)
2	13 (18)
No data	5 (7)
Smoking status	
Current smoker	31 (44)
Former smoker	29 (41)
Never smoked	11 (15)
Histology	
Adenocarcinoma	43 (60)
Squamous cell carcinoma	14 (20)
Large cell carcinoma	14 (20)
First-line treatment	
Cisplatin-gemcitabine	32 (45)
Carboplatin-gemcitabine	19 (27)
Cisplatin-pemetrexed	10 (14)
Carboplatin-pemetrexed	10 (14)
Second-line treatment	
Taxanes	11 (15)
EGFR TKIs	22 (31)
Others	9 (13)
Response rate	
Complete response	0 (0)
Partial response	27 (38)
Stable disease	26 (37)
Progressive disease	11 (30)
No data	7 (10)

Paraffin-embedded tumor specimens were collected before beginning chemotherapy and patients who had previously received radiotherapy were excluded. Tumor samples were evaluated by the pathologist to ensure a minimum of 90 % of tumor cells in analyzed samples. After mRNA extraction and retro transcription, the mRNA expression levels of *BRCA1*, *RNF8*, *UBC13* and *HERC2* were quantified using real-time PCR with a comparative method, as previously described [8]. mRNA levels were considered as categorical variables and dichotomized using the median value as cut-off point (Additional file 1: Supplementary Methods).

The median PFS and OS were estimated using the Kaplan-Meier method and compared with a two-sided log-rank test. Multivariate analyses were performed with the Cox regression method, with Eastern Cooperative Oncology Group (ECOG) performance status (PS) as covariate. All statistical analyses were performed using Statistical Package for Social Science (SPSS) for Windows version 17 (Chicago, IL, USA). Significance was set at *p* < 0.05.

## Results and discussion

The clinical features of the study population are summarized in Table 1. Patients were mainly male (76 %), asymptomatic or poorly symptomatic at diagnosis (ECOG PS 0–1, 75 %), and current or former smokers (85 %). The prevalent histology was adenocarcinoma (60 %). First-line treatment was chosen according to standard clinical practice and did not include taxanes or vinca alkaloids (Table 1).

The expression of each gene was successfully analyzed in 87-96 % of patients (Additional file 2: Table S1).

The median PFS for all 71 patients was 7.2 months (95 % confidence interval, CI = 5.8-8.5) and the median OS was 10.7 months (95 % CI = 9.2-12.2) (Additional file 3: Figure S1). No differences in PFS or OS were observed according to the individual expression levels of any of the four genes.

**Fig. 2:**
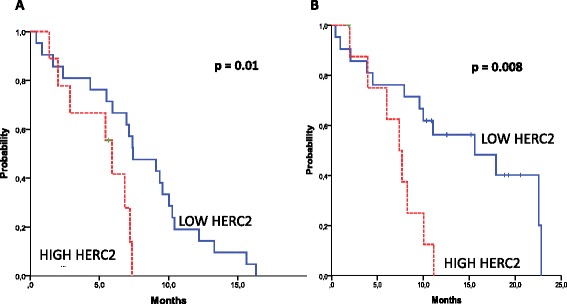
(**a**) Progression-free survival and (**b**) overall survival in 30 patients with low BRCA1 levels (lower than the median value) according to HERC2 levels

Based on our previous experience [23], we then examined the potential predictive value of a two-gene model and analyzed PFS and OS according to the combination of *BRCA1* with each of the three other genes. No significant differences were observed according to the combination of *BRCA1* and *UBC13* or of *BRCA1* and *RNF8* expression levels. However, the combination of *BRCA1* and *HERC2* identified subgroups of patients with different outcomes. Reliable quantification of mRNA was available for 55 patients. Among patients expressing low levels of *BRCA1*, the median PFS was 7.4 months (95 % CI = 4.5-10.4) for those expressing low *HERC2* levels, compared to 5.9 months (95 % CI = 4.8-7.1) for patients with high *HERC2* levels (*p* = 0.01) (Fig. 2a). The median OS was 15.3 months (95 % CI = 5.5-25.8) for patients expressing low *BRCA1* and *HERC2* and 7.4 months (95%CI = 5.1-9.7) for those with low *BRCA1* but high *HERC2* levels (*p* = 0.008) (Fig. 2b). Consistently, response rates were 42.9 % in patients expressing low levels of both *BRCA1* and *HERC2* and 28.6 % in those with low *BRCA1* but high *HERC2* levels. Progressive disease was recorded as best radiological response in 23.8 % and 42.9 % of patients, respectively (Additional file 4: Table S2). In contrast, *HERC2* expression levels did not affect the outcome of patients expressing high levels of *BRCA1* (Additional file 5: Figure S2).

To confirm the predictive value of this model, we assessed multivariate analysis and selected a more homogeneous population according to performance status (PS). Multivariate analyses showed that among patients with ECOG PS 0–1, high levels of either BRCA1 or HERC2 were associated with an increased risk of progression (HR, 1.4; 95 % CI = 1.1-4.73; *p* = 0.03) and death (HR, 3.7; 95 % CI = 1.54-9.1; *p* = 0.004) (Table 2), while among patients with PS 2, no significant differences according to BRCA1 and HERC2 levels were observed.

**Table 2:** Multivariate analyses of progression-free and overall survival in 53 patients with ECOG PS 0-1

Progression-free survival			
	Hazard ratio	95 % CI	*P*-value
Performance status			
0	1.00 (referent)		
1	1.45	0.62-3.41	0.39
BRCA1/HERC2 expression			
Low/Low	1 (referent)		
Other combinations	1.45	1.10-4.73	0.03
Overall survival			
	Hazard ratio	95 % CI	*P*-value
Performance status			
0	1 (referent)		
1	1.35	0.45-4.1	0.58
BRCA1/HERC2 expression			
Low/Low	1 (referent)		
Other combinations	3.70	1.54 -9.10	0.004

The identification of predictive markers of platinum sensitivity in lung cancer can help optimize the chemotherapy approach in advanced NSCLC patients. While a defective DNA damage repair capacity is associated with increased sensitivity to platinum, the translational application of this concept is rather difficult due to the complicated interplay among DNA repair pathways [13, 14]. The potential predictive roles of BRCA1 and excision repair cross-complementing 1 (ERCC1) have been widely studied in NSCLC [14, 24]. ERCC1 is a main component of NER, and low ERCC1 protein and mRNA expression have been associated with improved outcome in platinum-treated NSCLC patients, both in early-stage and in metastatic disease [14, 24]. However, neither BRCA1 nor ERCC1 expression was confirmed as a predictive marker in phase III studies [9, 25]. Nevertheless, our knowledge of DNA repair pathways is increasing, and a pivotal role has been demonstrated for post-translational modification in the response to double-strand breaks. In particular, the proteins UBC13 and RNF8, and more recently HERC2, are known to be essential for the recruitment of BRCA1 at DNA damage sites [13, 15–18].

The present study examined the hypothesis that the BRCA1 predictive model in advanced NSCLCs could be improved through the analysis of genes involved in the recruitment of BRCA1 at DNA damage sites through post-translational modifications (Fig. 1). In a retrospective, clinically homogeneous series of patients, we have demonstrated that *HERC2* mRNA expression provides additional predictive information when analyzed in conjunction with *BRCA1*. The outcome of patients expressing low *BRCA1* mRNA was significantly different according to *HERC2* mRNA expression and the median OS in patients expressing low levels of both *BRCA1* and *HERC2* was 15.3 months, which is greatly superior to that observed in unselected patients treated with platinum-based therapy (rarely exceeding 10 months) [26]. Moreover, a significantly increased risk both of progression and of death was associated with high expression of either *BRCA1* or *HERC2* with Cox regression analysis.

These findings lend support to the integrated analysis of several DNA repair components, which can provide enhanced results compared to the analysis of one component alone. Moreover, our results show the important role of HERC2 in determining platinum sensitivity in patients with low *BRCA1* expression, which is consistent with its known role in the recruitment of BRCA1 at DNA damage sites [18].

The main limitations of the study are its retrospective nature and the relatively small number of patients with mRNA expression data available for both *BRCA1* and *HERC2*. Due to the number of patients, we cannot draw definitive conclusions about the absence of predictive value of *UBC13* and *RNF8* in combination with *BRCA1* or of each gene when considered in isolation. In addition, mRNA expression data could not able to mirror the protein function and a validation through analyses of these markers at protein level could further increase the interest of these data. However, mRNA analysis is one of the most used surrogate marker of DNA repair capacity, taking into account the great difficulties in measuring DNA repair capacity at functional level. From the practical point of view, the need of highly experienced laboratories is one main limitation to the use of mRNA quantitative expression, while the possibility of testing more candidate markers is one strength point that could be particularly useful when studying a complex molecular pathway and the interplay of different components of the pathway.

Further development of this research would be the validation of the findings in a larger series of patients and the inclusion of a validation set of patients treated without platinum in order to test the hypothesis of a prognostic effect for the studied model. Finally the data should be confirmed prospectively.

## Conclusions

In our study population, integrated mRNA analysis of *BRCA1* and *HERC2* shows a predictive role in advanced NSCLC patients treated with first-line platinum-based chemotherapy. The patients expressing low levels of both genes achieved better outcome to platinum, while isolated analysis of *BRCA1* does not carry predictive value. Despite the limitations of the study, our results identify a new potential predictive marker, HERC2, and further refine the BRCA1 predictive model. Importantly, our findings support and pave the way for additional biomarker analyses, potentially useful for customizing chemotherapy in lung cancer.

### Ethics approval and consent to participate

Ethical approval has been obtained at each Institution participating in the trial. In particular, patients from Sant Pau Hospital, Alicante General Hospital and Cluzeau Hospital were enrolled in EURTAC clinical trail, all the included patients signed informed consent approved both by the coordinating center of the prospective trial and by each local ethics committee (Sant Pau Hospital, Alicante General Hospital, Cluzeau Hospital). The consent forms used for patients treated at the Istituto Oncologico Veneto were presented and approved by the Ethics Committee of Azienda Ospedaliera in Padova and the local Institutional Review Board (IRB) has reviewed the study and waived the need for formal ethic approval.

All the patients have signed a written informed consent form, approved at each local IRB.

## Additional files

Additional file 1: Supplementary Methods. Additional information on gene expression analyses. (DOCX 21 kb) Additional file 2: Table S1. Results of gene expression analyses in the study population (71 patients). (DOCX 11 kb) Additional file 3: Figure S1. The median progression-free and overall survival for all 71 patients. (DOCX 108 kb) Additional file 4: Table S2. Best radiological response in patients expressing low levels of BRCA1 according to HERC2 levels. (DOCX 10 kb) Additional file 5: Figure S2. The effect of HERC2 mRNA expression on the outcome of patients expressing high levels of BRCA1. (DOCX 198 kb)

## Abbreviations

BARD1: BRCA1-associated RING domain protein 1
BRCA1: breast cancer susceptibility gene 1
CI: confidence interval
ECOG: Eastern Cooperative Oncology Group
ERCC1: excision repair cross complementing 1
HERC2: HECT domain and RCC1-like domain-containing protein 2
HR: hazard ratio
MDC1: mediator of DNA damage checkpoint 1
NER: nucleotide excision repair
NSCLC: non-small-cell lung cancer
OS: overall survival
PFS: progression-free survival
PS: performance status
RAP80: receptor-associated protein 80
RNF8: RING finger ubiquitin ligase 8
UBC13: ubiquitin conjugating enzyme 13
