# Supplementary material for: Combinatory effect of BRCA1 and HERC2 expression on outcome in advanced non-small-cell lung cancer
Source: BMC Cancer. 2016 May 14;16:312. doi: 10.1186/s12885-016-2339-5 (PMC4868003; doi:10.1186/s12885-016-2339-5)
Supplement: Additional file 1: — Supplementary Methods. Additional information on gene expression analyses. (DOCX 21 kb) [file 12885_2016_2339_MOESM1_ESM.docx]

**Combinatory effect of BRCA1 and HERC2 expression on outcome in advanced non-small-cell lung cancer**

Laura Bonanno, Carlota Costa, Margarita Majem, Jose Javier Sanchez, Ignacio Rodriguez, Ana Gimenez-Capitan, Miquel Angel Molina-Vila, Alain Vergnenegre, Bartomeu Massuti, Adolfo Favaretto, Massimo Rugge, Cinta Pallares, Miquel Taron, Rafael Rosell

**Supplementary Material**

**Supplementary Methods**

**Gene expression analyses**

Gene expression profiling was performed using RNA isolated from the tumor tissue specimens, in accordance with a proprietary procedure of Pangaea Biotech SA. All samples were stained with haematoxylin/eosin and were analyzed by our pathologist before proceeding to micro- or macrodissection. All samples were processed with laser capture microdissection (Zeiss-Palm, Oberlensheim, Germany) or macrodissection to ensure a minimum of 90% of tumor cells per sample, thus avoiding contamination by non-tumoral cells. None of the samples obtained after micro- or macrodissection had more than 10% of lymphocytes, necrosis or stromal cells. After deparaffinization, the samples were lysed in a buffer containing tris-chloride, EDTA, sodium dodecyl sulphate (SDS) and proteinase K. RNA was than extracted with phenol-chloroform-isoamyl alcohol, followed by precipitation with isopropanol in the presence of glycogen and sodium acetate.

RNA was re-suspended in water and treated with DNAse I to avoid DNA contamination. cDNA was synthesized using M-MLV retrotranscriptase enzyme. Template cDNA was added to Taqman Universal Master Mix (Applied Biosystems [AB], Foster City, CA, USA) in a 12.5 µl reaction with primers and probes specifically designed for each gene using Primer Express 2.0 Software (AB). Gene expression was quantified using the ABI Prism 7900HT Sequence Detection System (AB). Dedicated primers and probes of beta-actin, BRCA1, RNF8, UBC13 and HERC2 were designed according to their Ref Seq in <http://www.ncbi.nlm.nih.gov/LocusLink>.

Relative gene expression quantification was calculated according to the comparative Ct method using β-actin as endogenous control and commercial RNA controls (UPE and Liver; AB and Stratagene, La Jolla, CA USA, respectively) as calibrators. Final results were determined as follows: 2-(ΔCt sample-ΔCt calibrator), where ΔCt values were determined by subtracting the Ct value of the target gene from the value of the β-actin gene. Gene expression analysis was always performed in triplicate, according to Technical Bulletin #2 (AB). The standard deviation (SD) of the Ct values was calculated, and the results were considered acceptable only when the SD was less than .30. When the SD was greater than .30, an additional independent analysis was performed. If the SD was greater than .30 in the second experiment, the sample was classified as non-evaluable.

All experimental procedures were carried out in accordance with the International Standard ISO 15189:2007(E) “Medical Laboratories” and “Particular requirements for quality and competence”. The Pangaea Biotech molecular biology laboratory is ENAC-accredited. We adhered to the MM16-A Volume 26 Number 29 CLSI Guideline “Use of External RNA Controls in Gene Expression Assays: Approved Guideline” and MM1-A2 Volume 26 Number 27 CLSI Guideline “Molecular Diagnostic Methods for Genetic Diseases; Approved Guideline-Second Edition”.
