# Supplementary material for: Combinatory effect of BRCA1 and HERC2 expression on outcome in advanced non-small-cell lung cancer
Source: BMC Cancer. 2016 May 14;16:312. doi: 10.1186/s12885-016-2339-5 (PMC4868003; doi:10.1186/s12885-016-2339-5)
Supplement: Additional file 2: Table S1. — Results of gene expression analyses in the study population (71 patients). (DOCX 11 kb) [file 12885_2016_2339_MOESM2_ESM.docx]

**Table S1.** Results of gene expression analyses in the study population (71 patients).

|  | **BRCA1** | **HERC2** | **RNF8** | **UBC13** |
| --- | --- | --- | --- | --- |
| Number of evaluable samples | 67 (94%) | 64 (90%) | 68 (96%) | 62 (87%) |
| Median expression level (range) | 13 (2-75) | 2.1 (0.4-15) | 8.2 (1-48) | 4.2 (1-22) |
