# Supplementary figures and images for: Combinatory effect of BRCA1 and HERC2 expression on outcome in advanced non-small-cell lung cancer
Source: BMC Cancer. 2016 May 14;16:312. doi: 10.1186/s12885-016-2339-5 (PMC4868003; doi:10.1186/s12885-016-2339-5)

**Figure S1.** The median progression-free and overall survival for all 71 patients.


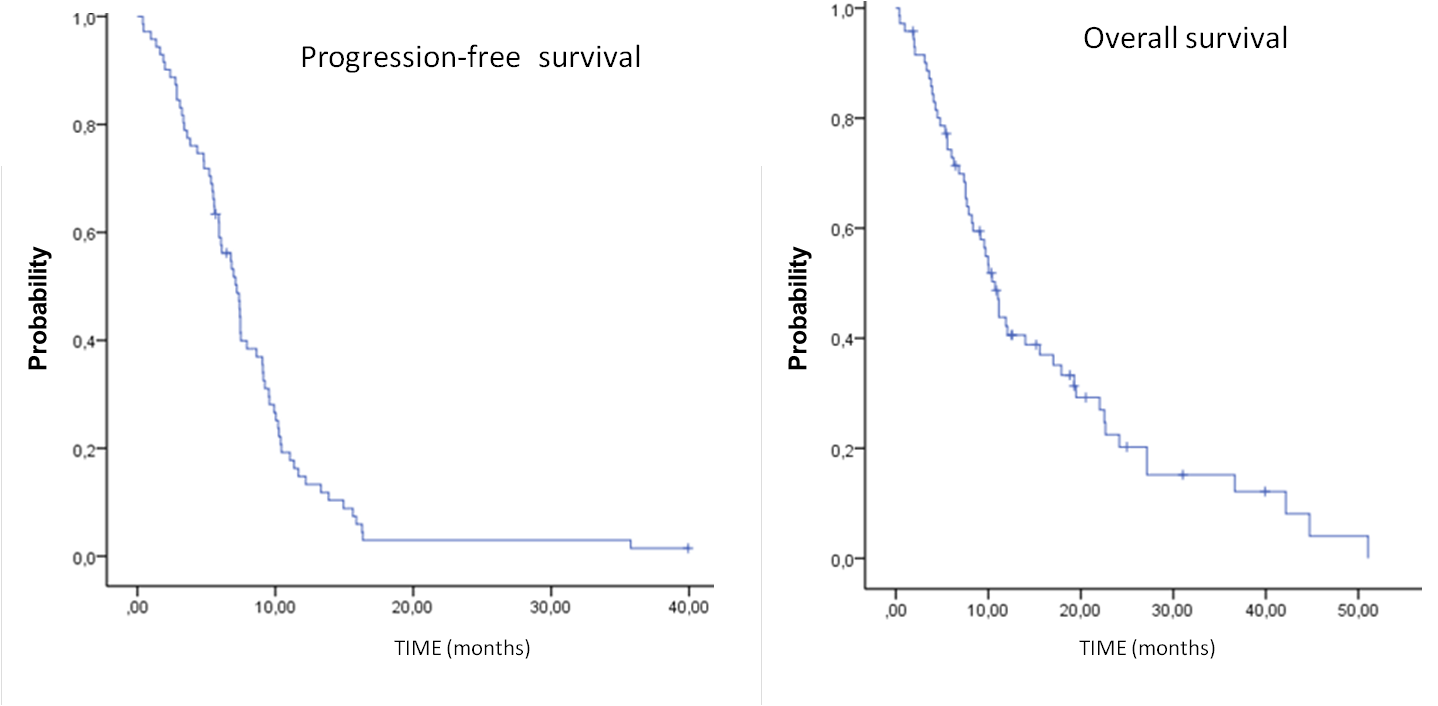

Supplement: Additional file 3: Figure S1. — The median progression-free and overall survival for all 71 patients. (DOCX 108 kb) [file 12885_2016_2339_MOESM3_ESM.docx]
