# Supplementary material for: Combinatory effect of BRCA1 and HERC2 expression on outcome in advanced non-small-cell lung cancer
Source: BMC Cancer. 2016 May 14;16:312. doi: 10.1186/s12885-016-2339-5 (PMC4868003; doi:10.1186/s12885-016-2339-5)
Supplement: Additional file 4: Table S2. — Best radiological response in patients expressing low levels of BRCA1 according to HERC2 levels. (DOCX 10 kb) [file 12885_2016_2339_MOESM4_ESM.docx]

**Table S2.** Best radiological response in patients expressing low levels of BRCA1 according to HERC2 levels.

|  | Low HERC2 expression  N (%) | High HERC2 expression  N (%) | p-value |
| --- | --- | --- | --- |
| Complete response  Partial response  Stable disease  Progressive disease  No data | 0 (0)  9 (42.9)  7 (33.3)  5 (23.8)  0 | 0 (0)  2 (28.6)  2 (28.6)  3 (42.9)  2 (28.6) | 0.61 |
