# Supplementary material for: Combinatory effect of BRCA1 and HERC2 expression on outcome in advanced non-small-cell lung cancer
Source: BMC Cancer. 2016 May 14;16:312. doi: 10.1186/s12885-016-2339-5 (PMC4868003; doi:10.1186/s12885-016-2339-5)
Supplement: Additional file 5: Figure S2. — The effect of HERC2 mRNA expression on the outcome of patients expressing high levels of BRCA1. (DOCX 198 kb) [file 12885_2016_2339_MOESM5_ESM.docx]

**Figure S2.** The effect of *HERC2 mRNA* expression on outcome of patients expressing high levels of *BRCA1*
